# RAGEISM: RACISM, AGEISM, AND THE QUEST FOR LIBERATION DEMOCRACY

**DOI:** 10.1093/geroni/igae098.0180

**Published:** 2024-12-31

**Authors:** Maya Rockeymoore Cummings

**Affiliations:** Johns Hopkins University, Baltimore, Maryland, United States

## Abstract

Structural discrimination subjects people from low-income and historically marginalized racial and ethnic groups in the United States to an anomalous aging process that exacerbates their exposure to social and environmental harms, deprives them of essential resources, and increases their levels of health-harming chronic stress. Although not typically referred to as such, this can also be considered a form of ageism. But the combination of racism and ageism, or “rageism,” should be considered a unique phenomenon because of the compounded effects of both forms of discrimination on the lives of those who experience it. In this formulation racism still means a system of oppression enacted against people with darker skin—and can be extended to the intersecting isms associated with class, gender, sexuality, etc.—but the term ageism, which typically refers to discrimination against older adults aged fifty and over, is redefined to describe the systematic effort to deprive black and brown people by deliberately sabotaging their quality of life at every stage of the life course. This reconceptualization helps us to understand age discrimination in its most complete and pernicious form. The official definition of rageism, therefore, is a systematic and persistent form of race, ethnic, and age-based discrimination that uses political institutions, public policy, and other levers of power to undermine the quality and length of life of low-income and non-European racial and ethnic groups from pre-birth to death and across generations. This talk will describe and provide examples of rageism while offering a framework to overcome it.

